# Supplementary material for: Fast delivery of heralded atom-photon quantum correlation over 12 km fiber through multiplexing enhancement
Source: Nat Commun. 2024 Nov 27;15:10306. doi: 10.1038/s41467-024-54691-3 (PMC11603145; doi:10.1038/s41467-024-54691-3)
Supplement: Supplementary file 1 — Supplementary Information [file 41467_2024_54691_MOESM1_ESM.pdf]

Supplementary Information: "Fast delivery of heralded atom-photon quantum correlation over 12 km fiber through multiplexing enhancement"

Sheng Zhang,[1,*] Jixuan Shi,[1,*] Yibo Liang,[1] Yuedong Sun,[1] Yukai Wu,[1,2] Luming Duan,[1,2,†] and Yunfei Pu[1,2,‡]

[1]*Center for Quantum Information, IIIS, Tsinghua University, Beijing 100084, PR China*

[2]*Hefei National Laboratory, Hefei 230088, PR China*

* These authors contributed equally to this work

† lmduan@tsinghua.edu.cn

‡ puyf@tsinghua.edu.cn

Supplementary Note 1. Experimental setup and method

In this section, we describe the experimental implementation of a spatially-multiplexed quantum memory array combined with angular degree of freedom. The experimental realization of a 2D quantum memory array with hundreds of individually addressable cells has been elaborated in our previous works [1, 2]. In this work, we integrate the angular degree of freedom into the spatially-multiplexed quantum memory array to enlarge the multimode capacity. When a memory cell is excited by the focused write beam, we collect signal photons from four symmetrical angles (labeled as A,B,C,D), as depicted in Supplementary Fig. 1a. The light emitted from the same cell at these four angles corresponds to distinct regions on the AOD crystal. We employ D-type mirrors to further seperate these four spatial patterns and direct them into four single-mode fibers with varying lengths, respectively. Subsequently, after passing through four fibers and four AOM switches, the four angular modes from one memory cell are transformed into four time-bin modes within the same fiber. Upon sequentially exciting all the memory cells in the 2D array (10×10), the 100 spatial modes and four angular modes are combined into 400 photonic time-bin modes, suitable for implementing multiplexing enhancement over a long-distance fiber.

In addition, we need each mode to have a sufficient lifetime to store the atom-photon correlation during the long-distance fiber transmission. The coherence time of each memory cell over $500\,\mu$s has been demonstrated in our prior works [1, 2]. Here we follow the similar methods to extend the coherence time, employing a collinear configuration and optical pumping to the clock state. However, due to utilizing four angular modes simultaneously, achieving complete collinearity between the write beam and signal photon is impossible. In this setup, the angle between the write beam and the signal photon is $0.3°$. The initial state $|g\rangle \equiv |5S_{1/2}, F=2, m=0\rangle$ is prepared after a $60\,\mu$s optical pumping process with a bias magnetic field of $0.5\,$G. As illustrated in Supplementary Fig. 2a, the whole optical pumping process consists of three parts. In the first $20\,\mu$s, we optically pump all the atoms to $|5S_{1/2}, F=1, m=+1\rangle$ state using three pump lights with different frequencies and polarizations, including one σ_+ light coupling the $|5S_{1/2}, F=1\rangle \rightarrow |5P_{1/2}, F=1\rangle$ transition, one π light coupling the $|5S_{1/2}, F=2\rangle \rightarrow |5P_{1/2}, F=2\rangle$ transition, and another σ_+ light coupling the $|5S_{1/2}, F=2\rangle \rightarrow |5P_{3/2}, F=2\rangle$ transition. In the second $20\,\mu$s, we execute a π flip between the $|5S_{1/2}, F=1, m=+1\rangle$ state and the $|5S_{1/2}, F=2, m=0\rangle$ state using a microwave transition at a specific frequency of $6.835003\,$GHz. In the final $20\,\mu$s, we apply a clean π pulse resonant to the $|5S_{1/2}, F=1\rangle \rightarrow |5P_{1/2}, F=2\rangle$ transition to remove the atoms remaining at $F=1$ after the microwave transition. The overall efficiency of preparing the atoms to the $|5S_{1/2}, F=2, m=0\rangle$ state is about 88%, including the fidelity of the initialization to the state $|5S_{1/2}, F=1, m=1\rangle$ of 93%, and the fidelity of the microwave pi pulse of about 95%. After optical pumping, the optical depth is about 5 in the center cell and about 3 in the edge cell. With those efforts, we successfully extend the coherence time of each memory mode to $200\,\mu$s level, which is sufficient for our next experiments.

Here we measure the coherence time T_{coh} by fitting the decay of signal-idler cross correlation with different storage time. In atomic ensemble system, one need to convert the spin-wave to idler photons for measurement. The decay in the fidelity of the entangled state mainly originates from the decay of the cross correlation, but in a slightly slower rate. Thus in atomic ensemble system, we usually use the $1/e$ decay time of the cross correlation as a lower bound of the coherence time T_{coh}. In Supplementary Fig. 1e we demonstrate the measurement of memory coherence time by fitting the decay of cross correlation $g_{s,i}$ with time, in a function $g_{s,i}(t) = 1 + Ce^{-t^2/\tau^2}$. The averaged memory coherence time $T_{\text{coh}} = 235\,\mu$s is the coherence time averaged over twelve different representative memory modes by the fitting of cross-correlation, as shown in Supplementary Fig. 1d.

We also characterize the crosstalk between different angular modes within a single memory cell. Here we choose the central memory cell $(103, 103)$ as the target cell, focusing both write beam and read beam onto this target cell with AOD addressing. We collect the coincidence counts between four signal modes $(i = \{A, B, C, D\})$ and four idler modes $(j = \{A, B, C, D\})$. As shown in Supplementary Fig. 1c, the obtained results demonstrate that the cross correlation between the signal mode i and the idler mode j is close to 1 when $i \neq j$, indicating that the crosstalk between different angular modes is effectively negligible. Except the potential crosstalk, we haven't observed any other added noise by using 4 angular multiplexing. The small crosstalk between different angular modes (A, B, C, D) origins from the mode overlap between the four angular modes for signal photons. In this work we use angular separation to resolve the four angular-multiplexed (k-vector) signal/idler modes. The angular separation between the adjacent modes is a couple of times larger than the divergence angle of each mode (the divergence of a Gaussian mode), so that we can pick out each mode without significant efficiency loss (their overlap is small). This is shown in the bottom left inset of Supplementary Fig. 1a, that at the idler AOD (where the angular distribution of each mode at the atoms is mapped to spatial distribution), the four k-vector idler modes can be clearly separated. However, the angular separation is not that large, so there's still some mode overlap (at the scale of 1%) between adjacent angular modes, which induces some mix and crosstalk between them. The angular separation cannot be too large in this experiment as a large angular separation are projected to large spatial separation at the position of the AOD, after the transformation of the three lenses (two of them are 150mm focus, and the other 50mm focus), as shown in the bottom left inset of Supplementary Fig. 1a. Larger spatial separation at the AOD will induce slower switching of the AOD to address different memory cells, which is not favorable. Thus here we find a point in this trade-off between crosstalk and switching time, to achieve a fast switching time of $1.7\,\mu$s and a crosstalk $< 1\%$ for the experiment.

However, in this work we do not use the angular separation to resolve the write/read laser from the signal/idler photon, as this angle separation of $0.3°$ is at the same level of the divergence angle of the signal/idler mode (the Gaussian radius is $60\,$um

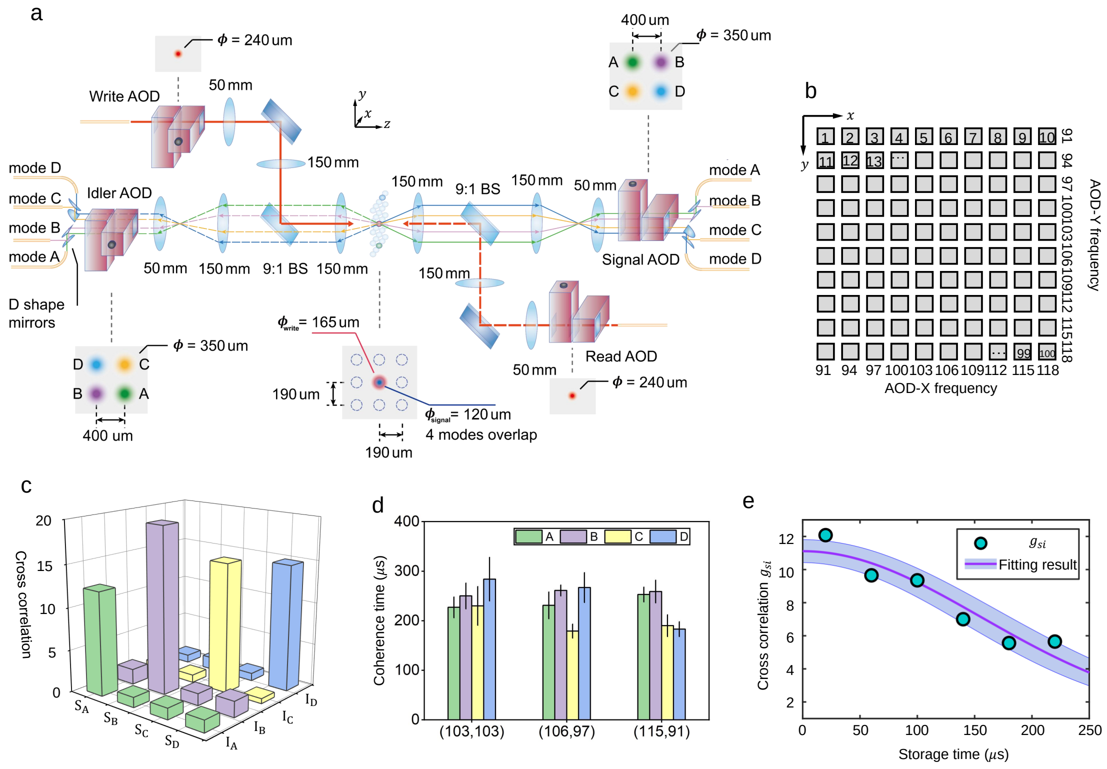

f

Signal

η_{BS}	η_{AOD}	η_{AOM}	η_{QFC}	η_{12km}	η_{SNSPD}	η_{other}	$\chi_{intrinsic}$	p_{suc}
90%	80%	85%	12%	59%	80%	80%	5.9%	0.16%

Idler

η_{BS}	η_{AOD}	$\eta_{pockels}$	$\eta_{etalons}$	η_{SPD}	η_{other}	$\eta_{intrinsic}$	η_{total}
90%	80%	83%	71%	50%	80%	15%	2.5%

Supplementary Fig. 1. **The hybrid-multiplexed quantum memory and performance.** **a**, Detailed experiment setup for the spatially-multiplexed quantum memory array combined with angular degree of freedom. **b**, This figure shows all the individually addressable memory cells in this work. The AOD addressing frequency for each memory cell is also demonstrated. **c**, The crosstalk between distinct angular modes in a single memory cell. Here the excitation probability is 0.2% and the storage time is $20\,\mu$s. **d**, Here we present the coherence time of 12 modes, which belong to three representative memory cells in Fig. 2b. The coherence time is fitted by cross correlation decay when varying storage time. **e**, Here we demonstrate how the memory coherence time of the mode in memory cell $(103, 103)$ and angular direction A is measured. Here we vary the memory storage time and measure the cross correlations. Then we fit the decay of cross correlation to a function $g_{s,i}(t) = 1 + Ce^{-t^2/\tau^2}$. The fitting gives a coherence time of $\tau = 227(21)\,\mu$s. **f**, Detailed lists of efficiencies of each optical component in the path of signal and idler channel. Error bars represent one standard deviation in this figure.

at the waist, thus a half divergence angle of roughly $0.25°$). Thus there is always substantial overlap between the write mode and the signal modes, no matter how long the path is. Thus we cannot resolve the write and signal via increasing the path length. In the experiment we combine and split the write/read laser with signal/idler mode by two 9:1 beamsplitters (with some sacrifice in efficiency), as illustrated in Supplementary Fig. 1a. In addition, it is not feasible to filter out the write/read laser from the signal/idler photon by angular separation in photon detection. Instead, we filter the unwanted write/read laser by spectrum method via three cascaded filter etalons. Each of the etalon can provide an extinction ratio of about 27 dB, thus 80 dB of extinction of the noise laser can be achieved. Together with another 20 dB from the polarization separation and another 10 dB from the angle (still 0.3 degrees separated), we can achieve a total extinction ratio of 110 dB to the write/read laser at the end, which is enough to eliminate the noise from write/read laser in both signal or idler photon.

The etalons for the detection of idler photons are customized fused silica solid etalons (Foctek Photonics), with a free spectral range of 13.6 GHz, and a finesse of about 30. The transmission efficiency for the signal/idler photon is about 90%, and the

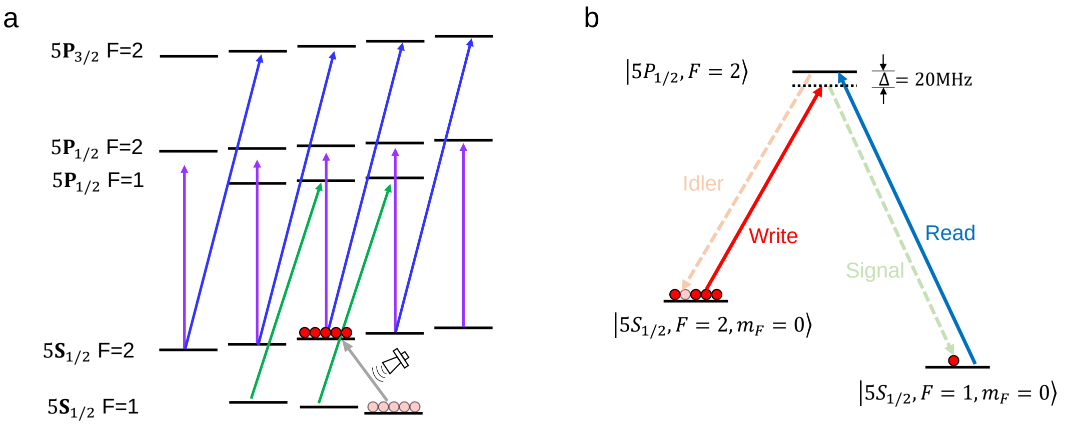

Supplementary Fig. 2. **Energy levels for optical pumping, write, and read.** **a**, The energy levels for the optical pumping process. **b**, The energy levels for the write process and read process.

elimination is roughly 27 dB for the write/read laser 6.8 GHz away. For frequency conversion, we use another customized air-spaced etalon (SLS optics) with ULE substrates, with a free spectral range of 10 GHz and a finesse of 150. The transmission efficiency is about 72%. All the etalons are in a plano-plano design. All the etalons in this experiment are actively stabilized by controlling the temperature of the homemade aluminum enclosure of the etalon, with a precision better than 0.01 K.

Supplementary Note 2. Implementation of the control logic

Now we describe the control logic employed to run the delivery of atom-photon quantum correlation over a 12 km fiber in a real-world application. To simulate this scenario, we utilize two optical fibers of equal length, as depicted in Fig. 1b. One is to transmit the converted 1546 nm signal photons to the SNSPD, while the other is used for transmitting the classic TTL signal back. This TTL signal serves as an heralding, which informs the memory whether the SNSPD successfully detects the incoming photon.

The experiment sequence begins with a 20 ms Magneto-Optical Trap (MOT) loading and a 2 ms molasses cooling, followed by 10 rounds of optical pumping, excitation, and heralding cycles. As illustrated in Supplementary Fig. 3, the arbitrary waveform generator (AWG) receives an initial trigger signal and subsequently outputs a series of pre-stored waveforms to drive write AOM, write AOD, signal AOD and signal AOMs. During the excitation stage (lasting 120 μs), the write beam sequentially visits all memory cells, while simultaneously 280 signal time-bin modes are directed into the telecom fiber. After a round-travel time of $2L/c = 120\,\mu$s, the first TTL signal returns, which is received by the Field-Programmable Gate Array (FPGA). During the heralding stage ($120\,\mu$s), the FPGA identifies the excited mode (both the cell and angle) based on the arrival time of the TTL pulse. Upon successful receiving of a TTL pulse, the corresponding index of the excited memory mode is mapped to switch signal to activate the Electro-Optic Modulators (EOMs) and an RF pulse with the corresponding frequency to drive the Read AOM, Read AOD, and Idler AOD. The entire process for mode identification and read out is carried out using a home-made FPGA, Digital-to-Analog Converters (DACs), and Voltage-Controlled Oscillators (VCOs). The two different read styles (corresponding to variable storage time and fixed storage time) can be easily switched by programming FPGA.

Supplementary Note 3. Different mode numbers in the 12 km fiber

Here we discuss the experimental sequences for changing the mode numbers in the 12 km fiber. As shown in Supplementary Fig. 4a, when only one memory cell is excited, the total heralding window (the time span during which heralding signals are expected) contains four photonic time bins. Due to the round-trip travel in two 12 km fibers, there exists a constant time difference between the excitation and the subsequent TTL heralding. If the number of modes is changed, the excited memory cells extend from the central region towards the exterior region of the memory array. The reason behind our selection of the lower-left to upper-right regions is for the minimal frequency variation after deflected by the signal AODs, which can cause efficiency variation in the transmission of a narrow etalon (FWHM$\sim$ 100 MHz) used for filtering out the pumping laser during the wavelength conversion.

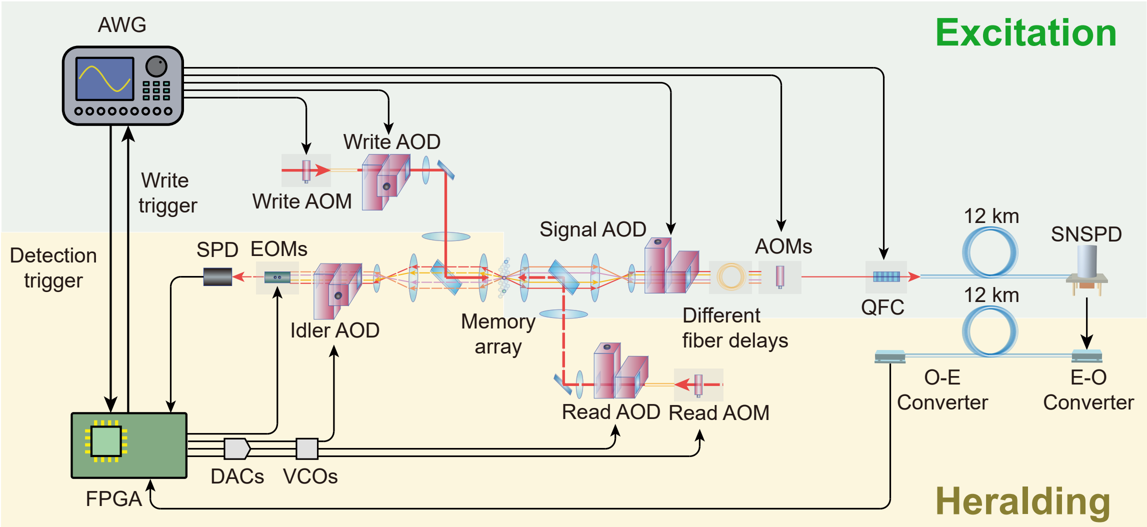

Supplementary Fig. 3. **The control system.** The upper part (light green background) includes all the optical and electronic components working during the excitation stage. The lower part (light yellow background) includes all the optical and electronic components working during the heralding stage. Once the MOT loading is finished, the sequential excitation is started.

The average storage time varies between the two read styles. The first read-out style (Fig. 4) is to immediately read the corresponding memory cell upon receiving the heralding signal. Consequently, the storage time for all the memory cells is the same. Taking into account the access time $10\,\mu$s required to write the DAC and drive the AOD in the read-out, the fixed storage time of all the memory cells is $\frac{2L}{c} + 10 = 130\,\mu$s. The second read style (Fig. 5) is to read when all the heralding signals have returned. In this case, the average storage time is dependent on the number of used modes. The minimum storage time is still $130\,\mu$s. The maximum storage time is $\frac{2L}{c} + \frac{N}{280}\frac{2L}{c} + 10 = (130 + 120\frac{N}{280})\,\mu$s if the number of total used modes is N. For example, if $N = 40$, the maximum storage time is $147\,\mu$s; if $N = 280$, the maximum storage time is $250\,\mu$s. In summary, the first read style ensures a fixed storage time for all memory cells, while the second read style results in a variable storage time based on the number of modes used.

Supplementary Note 4. Excitation of 400 modes locally

As a preliminary experiment, we demonstrate the sequential excitation of all memory cells in a 10×10 array without involving telecom conversion or the transmission through a long fiber. In the absence of the long fiber, the constraint of round-trip communication is eliminated. As illustrated in Supplementary Fig. 5a, the excitation window aligns with the detection window on the timeline. Given a switching time of $1.7\,\mu$s between different memory cells, the total scanning time for the 10×10 array amounts to $170\,\mu$s, which remains well below the coherence time. Consequently, we excite the entire 10×10 array, thereby generating 400 pairs of atom-photon correlations locally. Although there isn't a series of photonic time-bin pulses traveling through a long fiber, we are still able to record the arrival times of the 400 signal photons using the Single Photon Detector (SPD). The histogram in Supplementary Fig. 5b illustrates the distribution of the 400 time-bin modes. The excited memory mode is read out at the end of the $170\,\mu$s. By setting the success probability of each mode to 0.17%, the average cross-correlation across the 400 modes reaches 14.8 ± 1.7.

This preliminary experiment serves as a first step to test the multiplexed protocol for further experiment with longer fiber.

Supplementary Note 5. Delivery of atom-photon nonclassical correlation with 1km fiber

The signal photon at 795 nm has an attenuation about 4.0 dB/km in the single mode fiber used in our lab, thus can be transmitted with a fiber of $\sim$ 1 km. In our work, we also investigate atom-photon quantum correlations after the signal photons are transmitted through a 1 km fiber without wavelength conversion. The transmission time of the 1 km fiber is approximately 5 μs, and we use 12 modes in this experiment. Here no frequency conversion is used. Since the scanning time of three cells is

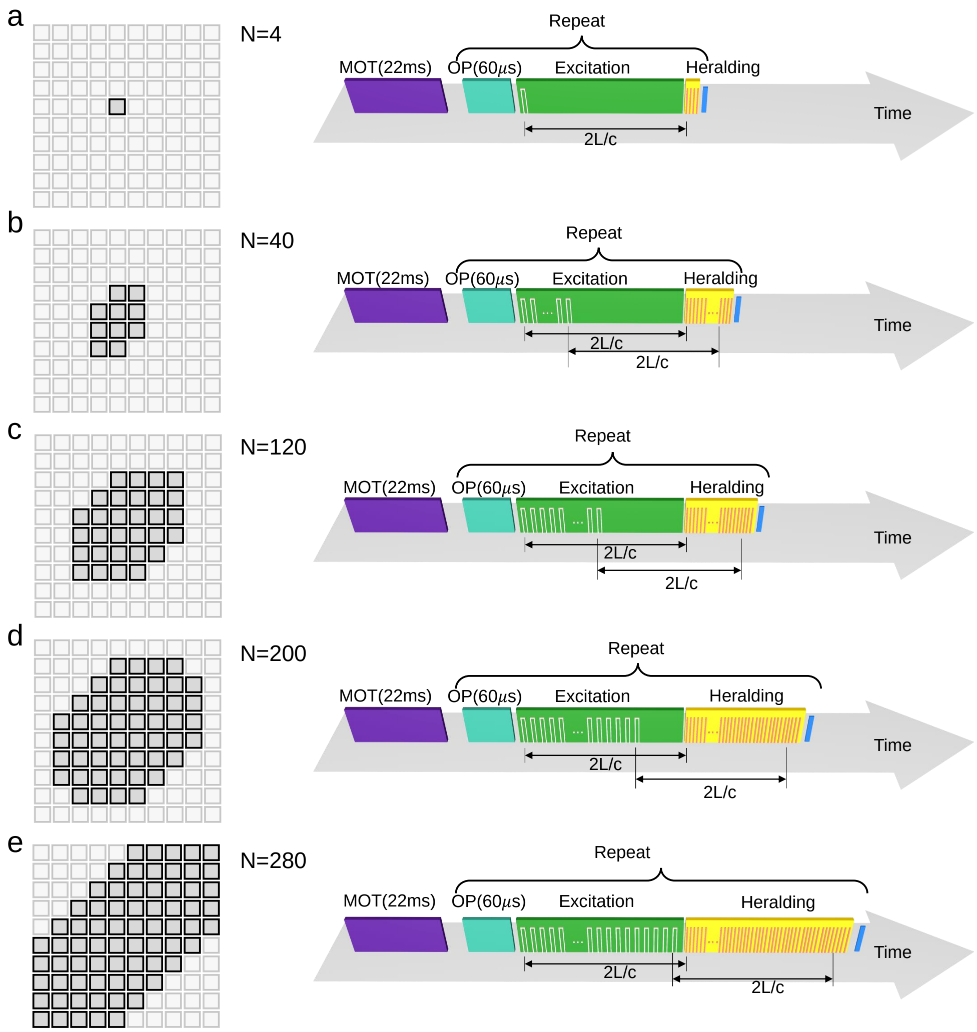

Supplementary Fig. 4. **Protocols for different mode numbers. a-e**, The selected memory cells and the experimental sequences when changing the memory mode numbers in the 12km fibers. The length of heralding stage varies with different mode number used.

shorter than switching time in read-out (10 μs), there is no significant difference between the two read styles. Note that here the heralding TTL is directly sent to the FPGA and does not experience the second transmission in the 1 km fiber, which is different from the stringent protocol demonstrated in main text. We identified the excited memory mode and retrieved the corresponding idler photon at the end of the detection window. In Supplementary Fig. 6c, we present the signal photons, idler photons, and coincidence counts for the 12 modes collected over 624 seconds. The average success probability of the 12 modes is 0.1%. The average cross-correlation across the 12 modes is 17.1 ± 1.6, with an average retrieval efficiency of 2.6%.

Supplementary Note 6. Evaluation of the cross correlation for the multimode quantum memory

Here we outline how to evaluate the average cross correlation over 280 modes, as shown in Fig. 4 and Fig. 5 of the main text. For a DLCZ-type quantum memory, the cross correlation is defines as $g_{s,i} = \frac{p_{s,i}}{p_s p_i} = \frac{p_{(s,i|s)}}{p_i}$, where $p_{(s,i|s)}$ is the conditional probability of detecting signal-idler coincidence given a signal is received. Here we also have $p_i = p_{(s,i|s)}p_s + p_{(\bar{s},i|\bar{s})}p_{\bar{s}}$, where $p_{(\bar{s},i|\bar{s})}$ is the conditional probability of detecting an idler photon during read-out given no signal photon is detected in write, and $p_{\bar{s}}$ denotes no signal photon is detected in write. As $p_s \approx 0.1\%$, $p_{\bar{s}} \approx 1$, and $p_{(s,i/s)}$ is only about ~ 10 folds larger than $p_{(\bar{s},i|\bar{s})}$, thus $p_i \approx p_{(\bar{s},i|\bar{s})}p_{\bar{s}}$, and $g_{s,i} = \frac{p_{(s,i|s)}}{p_i} \approx \frac{p_{(s,i|s)}}{p_{(\bar{s},i|\bar{s})}p_{\bar{s}}} \approx \frac{p_{(s,i|s)}}{p_{(\bar{s},i|\bar{s})}}$. If we get some signal photon clicks (S) after one round of excitation, we identify and retrieve the excited modes into idler photons to collect the corresponding coincidence counts (C). If no signal photon click is detected after one round of excitation, we record the number of these situations (N) and retrieve one

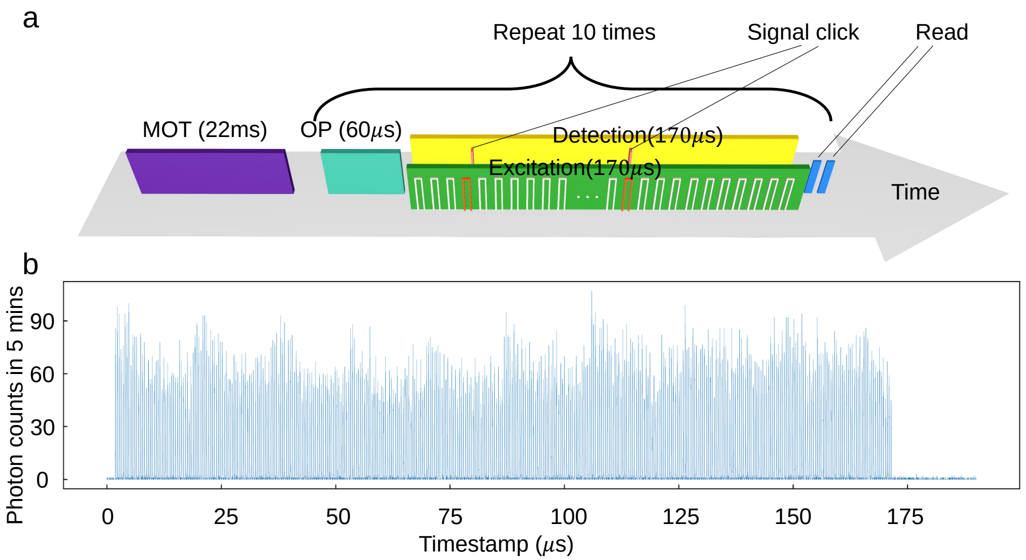

Supplementary Fig. 5. **Test experiment with** 400 **modes and short fiber.** **a**, The experimental sequence for excitation of 400 modes locally. **b**, The histogram of the 400 time bin modes recorded by the single photon detector.

mode randomly selected from all modes into idler photon and collect the idler photon click (I). The average cross correlation across all modes is then estimated to be $g_{s,i} = \frac{p_{(s,i|s)}}{p_{(\bar{s},i|\bar{s})}} = \frac{C/S}{I/N} = \frac{CN}{SI}$. The standard deviation is calculated assuming that all photon clicks follow the Poisson distribution.

Supplementary Note 7. Improve the performance of the multi-mode quantum memory in the future

In this section we demonstrate how to further improve the memory performance in the future. There are two main aspects which can be improved in the future to largely boost the performance of our multimode quantum repeater node, which include: (i) improvement on the current imperfect frequency conversion in both background noise and overall efficiency, and (ii) improvement on the intrinsic retrieval efficiency and memory lifetime by loading the atomic cloud into a two dimensional optical lattice array.

The first potential improvement lies in the quantum frequency conversion. The detailed optical setup of the frequency conversion used in this experiment is illustrated in Supplementary Fig. 7a. There is still room to improve the efficiency in the filtering optics, the device efficiency of the ppln waveguide, and by avoiding the use of the fiber jumpers and fiber Bragg grating. Compared to the state-of-the-art efficiency of 57% with a similar wavelength configuration, it is possible to achieve a 4-fold increase in the overall efficiency as the overall efficiency is 12% in our case. At the same time, the current background noise is about 200 counts per second. For the filtering, we use a bandpass filter of 5 nm transmission bandwith, a fiber Bragg grating of 10 GHz transmission bandwidth, and an etalon of 70 MHz transmission bandwidth. We find the current background noise can be suppressed by about 5 times with a slight change in the wavelength of the pumping laser in the future, as shown in Supplementary Fig. 7c. It is noteworthy that the background noise in the frequency conversion is a limit for the cross correlation in the experiment. With the high background noise, we need to use a high signal photon excitation probability to guarantee a high signal-to-noise ratio, which is a reason for the low cross correlation in this experiment. With the change of the pumping laser wavelength from 1636 nm to 1642 nm, and optimization on the total transmission and detection efficiency in the signal channel, we estimate a 10-fold improvement in the signal-to-noise ratio can be achieved. In this case, we can lower down the excitation probability to achieve a high cross correlation.

The second potential improvement lies in loading the atomic ensemble into a two dimensional optical lattice array. With the optical lattice, the motional dephasing can be largely suppressed, and it is not hard to improve the memory coherence time to several tens of milliseconds, which is 50 times longer than the current 235 μs. At the same time, with the significantly improved optical depth due to optical lattice and optimal wavepacket shaping [3], the intrinsic retrieval efficiency can be improved significantly. An intrinsic retrieval efficiency above 50% can be achieved with a moderate optical depth of around 30 [4]. In addition, it is possible to improve the overall path and detection efficiency of the idler photon from current 17% to 50%, by removing in-between fiber connections, using anti-reflection coating in all the optics, and upgrading etalon efficiency (co-propagating configuration is no longer needed with lattice thus less etalons are needed). Finally, by replacing our current silicon detector with

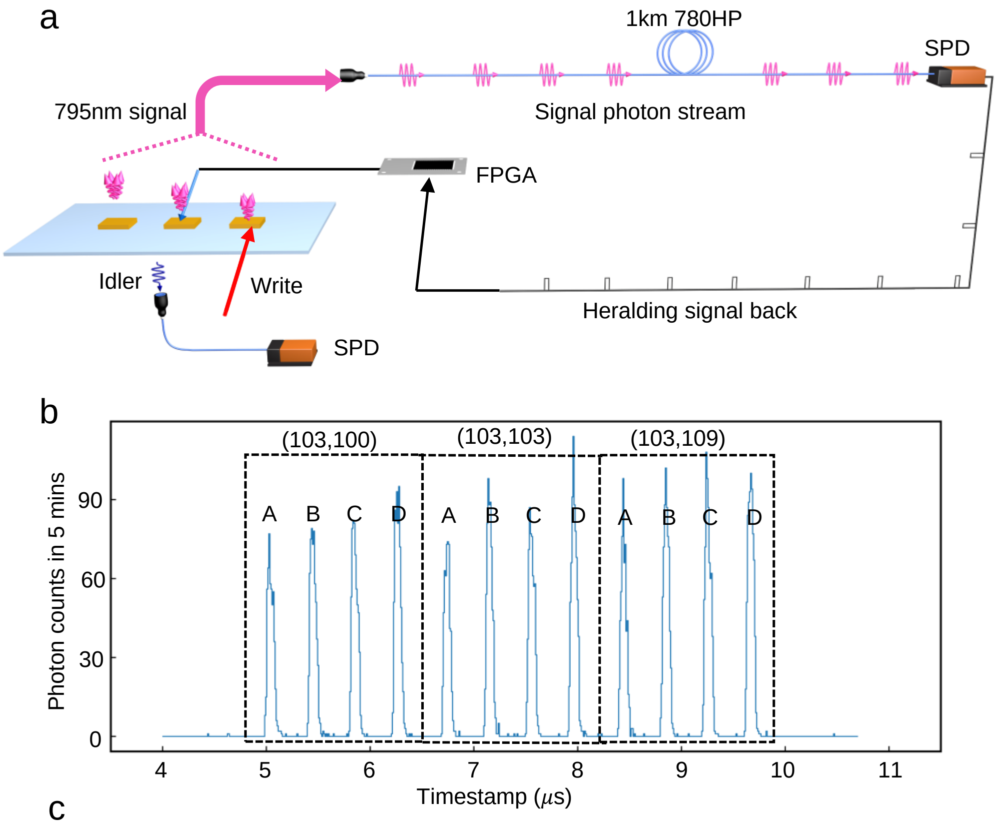

c

	Signal				Idler				Coincidence			
	A	B	C	D	A	B	C	D	A	B	C	D
(103,100)	345	498	450	426	57	46	59	60	9	6	11	14
(103,103)	365	511	452	449	40	40	73	64	14	5	12	14
(103,106)	402	559	457	505	58	39	81	74	16	9	11	22

Supplementary Fig. 6. **Delivery of atom-photon nonclassical correlation with 1 km fiber. a**, Schematic of signal photon transmission over 1km optical fiber. **b**, The histogram of 795nm signal photon. **c**, This table shows the signal photon, idler photon and coincidence counts of the 12 modes, collected in 624 s. The average success probability of the 12 modes is 0.1%. The memory array (containing 3 cells) is excited 454252 rounds, among which no signal photon is detected for 448848 times.

SNSPD, the detector efficiency can be improved to about 90%. With all these improvement, we can yield an overall retrieval efficiency of $50\% \times 50\% = 25\%$ in the future, which can significantly benefit the application such as entanglement swapping, and improve the cross correlation.

With all these potential improvements in the future, the performance of the multimode quantum repeater node demonstrated in this work will have a significantly boosted performance. With the improvement of intrinsic retrieval efficiency, if we set the intrinsic excitation probability χ to around 1% (currently 5.9%), we estimate a $g_{si} = 50$ can be achieved. At the same time, taking into account the improvement in the frequency conversion, optical path, detector, as well as the decrease in the excitation probability, we can achieve an expected entanglement generation time $T_{\text{ent}} \approx 200\,\mu$s, and a link efficiency $T_{\text{coh}}/T_{\text{ent}} = 10\,\text{ms}/200\,\mu\text{s} = 50$ can be achieved in this ideal case. The signal-to-noise ratio of the signal photon can also be improved to 50 in this case.

Supplementary Note 8. Phase stabilization in the atom-atom entanglement via single photon interference and different entanglement swapping methods

Here we show the scheme for combining two atom-photon quantum correlations demonstrated in this paper to generate heralded atom-atom entanglement via single photon interference in the future. Single photon interference have been employed

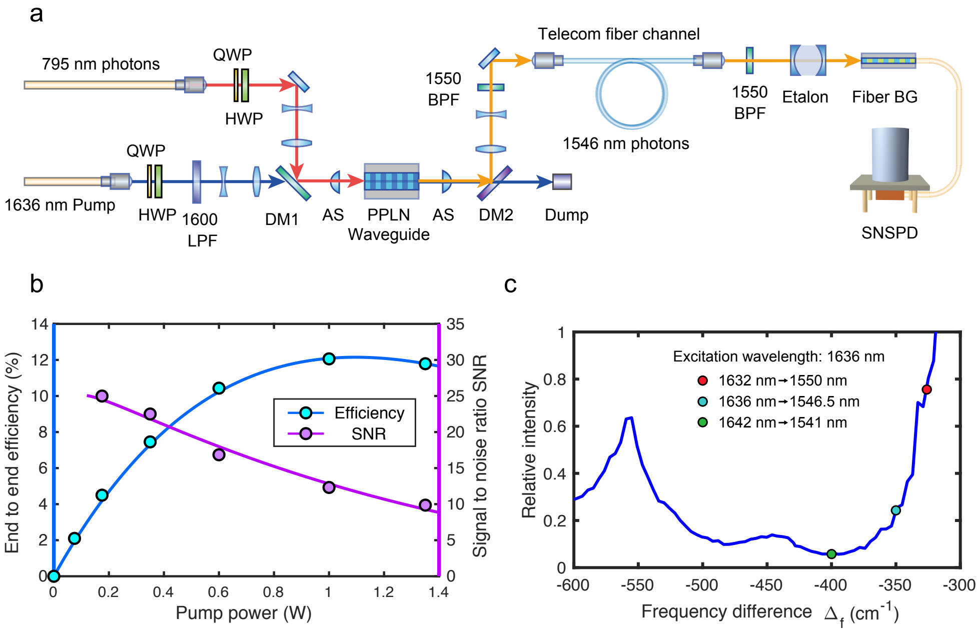

Supplementary Fig. 7. **Performance of the quantum frequency conversion.** **a**, The detailed setup for the frequency conversion and filtering module. **b**, The measured overall efficiency (blue) and the signal-to-noise ratio (purple) due to strong pumping laser as a function of the power of pumping laser, at an intrinsic probability of $\bar{\chi} \approx 0.8\%$. The signal is the detector clicks induced by signal photon in the detection window, and the noise includes the detector clicks in the detection window induced by the noise photon in the frequency conversion stage, the dark count of the SNSPD, and all other sources of noise photon. In the experiment we set the power to 1 Watt. **c**, The noise intensity in frequency conversion as a function of the frequency difference between the converted photon and the pumping laser, under the same pumping laser power. We fix the incoming signal to 795 nm, vary the pumping laser by three different wavelengths 1632 nm (red point), 1636 nm (blue point, used in this experiment), and 1642 nm (green point), and measure the noise spectrum by scanning the transmission peak of the fiber Bragg grating. We find that the noise spectrum versus frequency difference $\Delta_f = f_{\text{after conversion}} - f_{\text{pumping}}$ is very similar to the three pumping wavelengths, and here we plot the relative noise intensity as a function of Δ_f averaged over three different pumping wavelengths as the blue curve. Here we find that, by slightly adjusting the pumping laser from the current 1636 nm to 1642 nm, we can have a noise about only 20% of the current value, and the conversion efficiency is approximately the same as in the current case. This can be a scheme to further suppress the noise in frequency conversion in the future.

to generate atom-atom entanglement in many different systems [5–7]. Here we use a similar method as [6] to generate and verify the atom-atom entanglement with each node located in remote places.

The setup is demonstrated in Supplementary Fig. 8. Following the scheme described in the supplemental material of [6], here the generated atom-atom entanglement can be measured with an auxiliary EP (entanglement probe) pulse on each side. The auxiliary EP pulses can be regarded as another pair of idler-idler entanglement $e^{i\phi^A_{EP}}|01\rangle_{AB} + e^{i\phi^B_{EP}}|10\rangle_{AB}$, in the weak power limit (A/B represents different repeater node). The EP pulse and the idler photon are mixed in a PBS at each node for the entanglement measurement, as shown in Supplementary Fig. 8a. After the mix, the final idler-idler (atom-atom) entanglement can be expressed as a polarization maximally entangled (PME) state,

$$|\Psi^{\pm}_{\text{PME}}\rangle = \frac{1}{\sqrt{2}}(|HV\rangle_{AB} \pm e^{i\phi_{\text{PME}}}|VH\rangle_{AB}) \quad (1)$$

Here ϕ_{PME} is the phase of the PME state, which can be decomposed into two parts,

$$\phi_{\text{PME}} = \phi_{\text{lo}} + \phi_{\text{rm}} \quad (2)$$

Here $\phi_{\text{lo}} = \phi^A_{\text{lo}} - \phi^B_{\text{lo}}$ is the phase regarding local interferometers, with $\phi^{A/B}_{\text{lo}}$ represents the phase difference of two arms in the local interferometer at repeater node A or B, as illustrated in Supplementary Fig. 8a. ϕ_{rm} is the phase regarding the remote paths for the single photon interference, which includes the phase from the frequency conversion and the long fiber, as shown in Supplementary Fig. 8b. Thus the phase stabilization of the future atom-atom entanglement experiment involves the active stabilization of two different phases, which include the stabilization of two local interferometers, and the stabilization of the

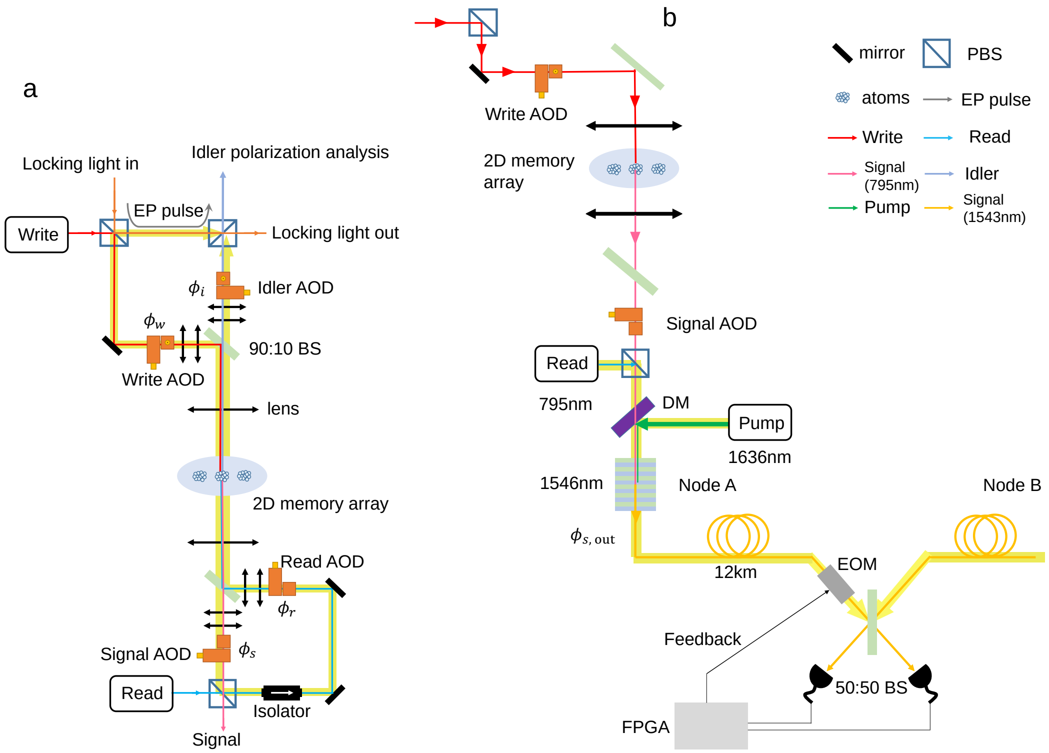

Supplementary Fig. 8. **Phase stabilization in atom-atom entanglement via single photon interference.** **a**, Detailed setup and the locking of the local interferometer (highlighted in yellow) in each repeater node. **b**, The generation of atom-atom entanglement between repeater node A and B through single photon interference. The stabilization of the remote paths between two repeater nodes are highlighted in yellow.

remote interference paths including two long fibers (12 km).

The first task is the stabilization of the local interferometers at both repeater node A and B, as shown in Supplementary Fig. 8a, following the scheme described in [6]. Here $\phi_{\mathrm{lo}}^A = \phi_w^A + \phi_r^A + \phi_s^A + \phi_i^A - \phi_{\mathrm{EP}}^A$ is the local interferometer highlighted in Supplementary Fig. 8a, where ϕ_w is the optical path phase of the write pulse, ϕ_r is the optical path phase of the read pulse, ϕ_s is the optical path phase of the signal photon, ϕ_i is the optical path phase of the idler photon, and ϕ_{EP} is the optical path phase of the EP pulse. Thus we can lock the interferometer highlighted in Supplementary Fig. 8a in each node, to achieve a stable local phase $\phi_{\mathrm{lo}}^{A/B}$.

The second task is the stabilization of ϕ_{rm}, which is the phase regarding two remote paths for single photon interference, as shown in Supplementary Fig. 8b. Similar to [6], this task is for stabilizing the relative phase experienced by the signal photons after leaving the local interferometer. Note that the phase from the pumping laser and frequency conversion module have been included in this ϕ_{rm}. In this case, there is no closed interferometer as in the local case, and the major phase drift are originated from the optical phase of the read and pump laser, as well as the drift of the long fiber (12km) [6, 8]. To stabilize this relative phase between two remote interference paths, one needs to send attenuated phase probe pulses into the long channels from both sides, and measure the photon counts after these two pulses interfering at the beamsplitter in the middle detection station, to evaluate the relative phase between the two long channels, before the entangling attempts. The feedback signal is applied to an EOM (see Supplementary Fig. 8b) to keep the phase ϕ_{rm} to a constant value. In this task, the local laser (write, read and pump) should be locked to a high-finesse and low-drift reference cavity to achieve a good laser linewidth and good stability. Frequency difference between lasers at different nodes also need to be measured and controlled to guarantee the frequency difference is small [6, 8]. With this method, the phase between the two remote path can be stabilized, similar to [6, 8].

Finally, with the stabilization of both the local phase ϕ_{lo} and the remote phase ϕ_{rm}, the atom-atom entangled state (Supplementary Eq. (1)) can be faithfully generated and verified.

In our multi-mode quantum repeater experiment, except the phase locking regarding a single mode described in Supplementary Fig. 8, another complexity originated from the spatial and angular multiplexing should also be considered. Here we analyze the phase stabilization when both of these two multiplexing methods are involved. The first issue is the phase stabilization of the spatially multiplexed modes involving different deflection angles on the AOD thus different spatial paths. Thanks to the passive stability of the AOD scanning system (because all optical paths go through the same optical elements), the phase difference between different paths (spatial modes) does not vary with time ($\Delta\psi_{i,j} = \psi_i - \psi_j = C_{i,j}$, $\Delta\xi_{i,j} = \xi_i - \xi_j = C'_{i,j}$, with $1 \le i, j \le n$, $C_{i,j}$ and $C'_{i,j}$ do not vary with time. n is the number of spatial modes, as shown in Supplementary Fig. 9) on the

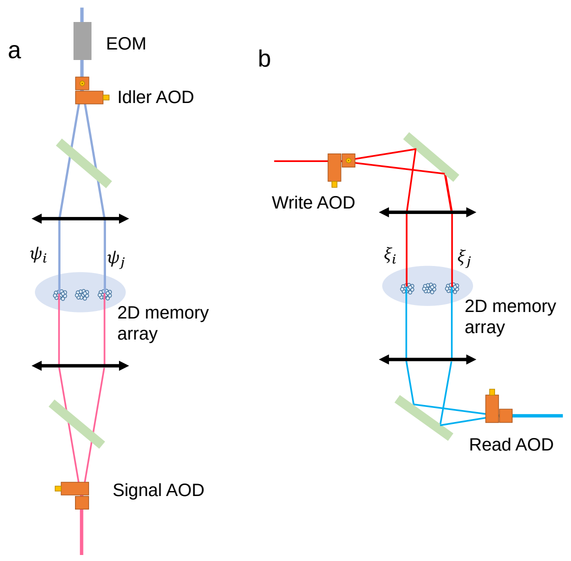

Supplementary Fig. 9. **Passive phase stabilization between different spatial modes.** **a**, ψ_i represents the total optical phase experienced by the signal path (purple) and idler path (blue) between the signal and idler AODs, for the ith spatial mode. **b**, ξ_i represents the total optical phase experienced by the write (red) and read (blue) lasers between write and read AODs, for the ith spatial mode.

scale of days [2, 9]. Note that for the spatial multiplexing, the potential time variation in the final phase can only happens on the paths between the AODs, which is $\Delta\psi_{i,j} + \Delta\xi_{i,j} = C_{i,j} + C'_{i,j}$ for any pair of i, j, as other optical paths are overlapped for ith and jth modes and are actively locked in the local interferometer as shown in Supplementar Fig. 8. Since $\Delta\psi_{i,j} + \Delta\xi_{i,j}$ is also a constant value due to passive stabilization, the phase difference between any pair i, j of the spatial modes does not vary with time. Thus we only need to apply the stabilization of local loop to one of the n modes, with all other modes automatically stabilized. Meanwhile, although the phase differences are constant between each pair of the paths, they are not zero. Thus careful calibration of the phase differences of all modes is needed before the experiment and real-time phase compensation can be applied after the heralding of atom-atom entanglement, according to which mode is entangled through the heralding signal arrival time. The real-time compensation can be executed by an EOM inserted into the idler photon path, as shown in Supplementary Fig. 9.

Another potential problem in atom-atom entanglement is that, in our current configuration, the signal photons from different spatial modes have slightly different frequencies on a scale of several to tens of MHz, which can cause phase difference in the long fiber if the fiber length of the both sides are not exactly the same. We can solve this problem by precisely controlling the fiber length difference between both sides, or just by compensating the frequency difference of signal photon from each mode to the same value by an additional double-pass AOM right after the AOD (before the frequency conversion module). Another method to solve this problem is that we can use the crossed AOD scheme widely used in trapped-ion system [10], which is by using a pair of AODs to scan one dimension, thus the frequency difference between each mode is vanished. In this way we need four AODs for two dimensional scanning but the frequency difference problem between each spatial mode will no longer exist.

Regarding the overlapping of the wavepackets of the two signal photons from each node, we can achieve a wavepacket shape mismatch of 1 or 2 nanoseconds by adjust the difference of the two optical path lengths, following the implementation demonstrated in the supplementary information of [11]. This mismatch is small, considering that the wavepacket length of our signal photon is about 100 ns. The synchronization of the remote control systems has also been demonstrated in [6, 12].

Finally, we demonstrate how to stabilize the four angular modes. The stabilization of the four angular modes needs the stabilization of three signal-idler path interferometers (with one of the four modes already stabilized in the local loop in Supplementary Fig. 8). Thus we can actively stabilize the relative phase of three interferometers by three piezos as shown in Supplementary Fig. 10.

Here we also discuss the repeater performance with two different entanglement swapping methods. In the first swapping method, the ith elementary memory-memory entanglement in one segment can only be connected with ith elementary memory-memory entanglement in the neighboring segment. This is equivalent to n parallel single-mode quantum repeater running at the same time, where n is the total mode number in each multi-mode quantum repeater node. Thus this scheme is also called 'parallel scheme' as described in detail in [13].

In the second swapping scheme, the ith memory-memory entanglement pair can be connected with jth memory-memory entanglement pair, where i and j do not need to be same ($1 \leq i, j \leq n$, n is the mode number). This swapping scheme is

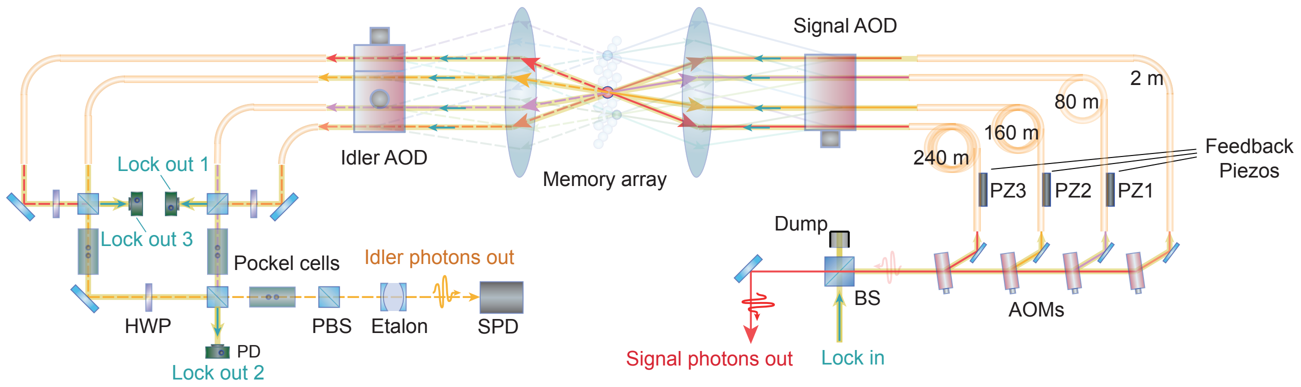

Supplementary Fig. 10. **Phase stabilization between different angular modes.** The relative phases between the four angular modes can be stabilized by three interferometers. We send a lock laser from the signal side which is split into four paths by switching AOMs, and lock out signals are detected at the idler side. Three interference loops are enough to lock the relative phase of the four modes. The feedback to lock the relative phases between different angular modes is implemented by three piezos stretching the fibers. Here by detecting the light of Lock out 1 and feedback to PZ1 we can lock the relative phase between the 2m (mode A) and 80m (mode B) paths; by detecting the light of Lock out 2 and feedback to PZ2 we can lock the relative phase between the 80m (mode B) and 160m (mode C) paths; by detecting the light of Lock out 3 and feedback to PZ3 we can lock the relative phase between the 160m (mode C) and 240m (mode D) paths.

called as ‘multiplexed scheme’ in [13], and can be supported by the random-access ability of our memory as demonstrated in the second readout style.

It is shown that the ‘multiplexed scheme’ has significant advantages in performance over ‘parallel scheme’, in several different scenarios, as illustrated in [13]. The advantage in efficiency is even larger in short coherence time case (low link efficiency case). Except the improvement in the efficiency, by employing ‘multiplexed scheme’ one can produce many copies of memory-memory entangled pairs in a segment, which can be used in case of failed entanglement swapping or further for entanglement distillation.

Supplementary Note 9. Entanglement fidelity vs cross-correlation for remote atom-atom entanglement and three-node quantum repeater in the future

In this section, we analyze the relation between cross-correlation g_{si} and the entanglement fidelity of potential atom-atom entanglement with several possible schemes. In the DLCZ quantum repeater protocol, the hamiltonian for the excitation process is [14, 15],

$$H = \Omega S^\dagger a^\dagger + h.c. \tag{3}$$

Here Ω is the coupling coefficient depending on the intensity and frequency of write laser, $S^\dagger$ is the creation operator of collective spin wave mode, $a^\dagger$ is the creation operator of signal photon, and $h.c.$ is the hermitian conjugation. The signal-photon spin-wave entangled state can be expressed as

$$|\psi_{s,a}\rangle = e^{-iHt_e}|00\rangle = (1 - \frac{1}{2}(\Omega t_e)^2)|00\rangle - i\Omega t_e|11\rangle - (\Omega t_e)^2|22\rangle + O((\Omega t_e)^3) \tag{4}$$

in case of small excitation, where t_e is the time of the excitation pulse (write). The excitation amplitude of $|11\rangle$ is $-i\Omega t_e$, corresponding to an intrinsic excitation probability of $\chi = (\Omega t_e)^2$. The probability of double excitation is $\chi^2 = (\Omega t_e)^4$.

After heralded signal photon is detected, the stored spin wave is then converted to an idler photon after a storage time of t. The cross correlation function $g'_{si}(t)$ of signal photon and idler photon before frequency conversion can be expressed as

$$g'_{si}(t) = \frac{p_{si}}{p_s p_i} = \frac{\chi\eta(t)\eta_s\eta_i + p_s p_i}{p_s p_i} = \frac{\chi\eta(t)\eta_s\eta_i + \chi\eta_s\chi(1+\eta(t))\eta_i}{\chi\eta_s\chi(1+\eta(t))\eta_i} = 1 + \frac{\eta(t)}{\chi(1+\eta(t))} \tag{5}$$

Here $p_s = \chi\eta_s$, $p_i = \chi(1+\eta(t))\eta_i$, p_{si} is the probability of detecting a signal photon, an idler photon, or a signal-idler coincidence per trial. $\eta(t)$ is the intrinsic retrieval efficiency in the read process after a storage time of t, which decays with time due to the finite lifetime of spin wave. η_s and η_i are the overall transmission and detection efficiencies in signal and idler channels.

In the experiment, due to the noise in frequency conversion, the measured cross correlation after the conversion can be expressed as [16]

$$g_{si} = \frac{g'_{si} + \text{SNR}^{-1}}{1 + \text{SNR}^{-1}} \tag{6}$$

Here g_{si} is the measured cross correlation after the frequency conversion, and SNR is the signal-to-noise ratio of signal photon after frequency conversion.

In our experiment, the intrinsic retrieval efficiency $\eta(t)$ is about 15% at $t = 0$, but decays to 11% at $t = 130\mu s$ (in fixed storage time mode) and 7.8% with an average storage time of $t_s = 190\mu s$ (in on-demand mode), suffering from the memory lifetime. Then the estimated intrinsic excitation probability is $\chi \approx 5.9\%$ when $g_{si} = 2.67$ in fixed storage time mode and $\chi \approx 3.9\%$ when $g_{si} = 2.7$ in the on-demand mode, based on Supplementary Eq. (5) and Eq. (6). This intrinsic excitation probability χ also determines the amount of multiple excitation in the atom-photon correlation and further atom-atom entanglement. A detailed calculation of the multiphoton error of DLCZ scheme can be found in [15]. Here the density matrix of the heralded atom-atom entanglement in two remote memories after successful heralding by single photon interference is

$$\rho_{a,a} = \frac{1}{1+\text{SNR}^{-1}}(|\Psi\rangle\langle\Psi| + [\frac{\chi}{2}(|20\rangle\langle 20| + |11\rangle\langle 11| + |02\rangle\langle 02|) + \frac{\chi}{2\sqrt{2}}(|20\rangle\langle 11| + |02\rangle\langle 11| + H.c.)]) + \frac{\text{SNR}^{-1}}{1+\text{SNR}^{-1}}\rho_{\text{noise}} + O(\chi^2) \tag{7}$$

$$= \rho_1 + \rho_2 + \rho_{\text{N}} + \rho_{>2} \tag{8}$$

where the first term of $|\Psi\rangle = \frac{1}{\sqrt{2}}(|01\rangle + |10\rangle)$ is the target entagled state, and the rest terms are the multiple excitation terms. Since our intrinsic excitation probability χ is at the level of several percents, the influence of higher terms $\rho_{>2}$ can be neglected. ρ_{noise} is the density matrix of a fully mixed state representing the incoherent noise, which has a fidelity of $\frac{1}{4}$, and $\rho_{\text{N}} = \frac{\text{SNR}^{-1}}{1+\text{SNR}^{-1}}\rho_{\text{noise}}$. The phases involved are set to 0 for simplicity.

Here we analyze the fidelity of remote atom-atom entanglement with several different schemes. In DLCZ protocol, we need to convert the atomic state (spin wave) to idler photon for further measurement. Here we consider three different schemes to generate and verify heralded atom-atom entanglement from a pair of atom-photon correlation demonstrated in this work. In the first scheme illustrated in Supplementary Fig. 11a, when a signal photon is recorded after the interference of signal photons from each node on a beamsplitter, the two remote memories are established into a Fock-state entanglement $|\Psi\rangle = \frac{1}{\sqrt{2}}(|10\rangle_{AB} + |01\rangle_{AB})$. For the verification of the atom-atom entanglement, one needs to convert the atomic state into idler photons at both nodes A and B, and interfere the idler photons from both sides on another beamsplitter, as demonstrated in [11, 17]. In this case, to verify the atom-atom entanglement, one need to stabilize the relative phase of the two long channels of idler photons from both sides, which adds significant difficulties to the experiment.

In the second scheme, one can combine an idler-idler Fock-state entanglement with two local weak coherent pulses, into a polarization maximally entangled state (PME), which has been demonstrated experimentally in [6], and also illustrated in Supplementary Fig. 8 and Supplementary Fig. 11b. In this case, the entanglement fidelity can be measured in a standard way. The idler photon entangled state from two ensembles can be expressed as $|\Psi\rangle = \frac{1}{\sqrt{2}}(|HV\rangle_{AB} + |VH\rangle_{AB})$ after the combination. Here we consider the infidelity of noise and multi-excitation. At the same time, the double excitation has also the doubled chance of being detected over single excitation on the photon detector.

In case of the EP pulse is very weak, the effective idler-idler density matrix can be expressed as

$$\rho_{i,i} = (1 - 3\chi)\frac{g_{si} - 1}{g_{si} + 1}|\Psi\rangle\langle\Psi| + (\frac{2}{g_{si} + 1} + 3\chi\frac{g_{si} - 1}{g_{si} + 1})\rho_{\text{noise}} \tag{9}$$

The fidelity of such a state to $|\Psi\rangle$ is

$$F = Tr(|\Psi\rangle\langle\Psi|\rho) = 1 - \frac{3}{2g_{si} + 2} - \chi\frac{9(g_{si} - 1)}{4g_{si} + 4} \tag{10}$$

as shown in Supplementary Fig. 11d.

The third scheme for generating and verifying atom-aotm entanglement is to combine a pair of remote Fock-state idler-idler entanglements on two PBSes located in each node to generate a polarization entangled state, as shown in Supplementary Fig. 11c, and previously demonstrated in [18]. In the case of receiving an idler photon at both A and B, the ideal idler-idler entangled state $|\Psi\rangle = \frac{1}{\sqrt{2}}(|HV\rangle_{AB} + |VH\rangle_{AB})$ can also be achieved. After taking the noise and multi-excitation error into account, the effective density matrix after heralded entanglement becomes

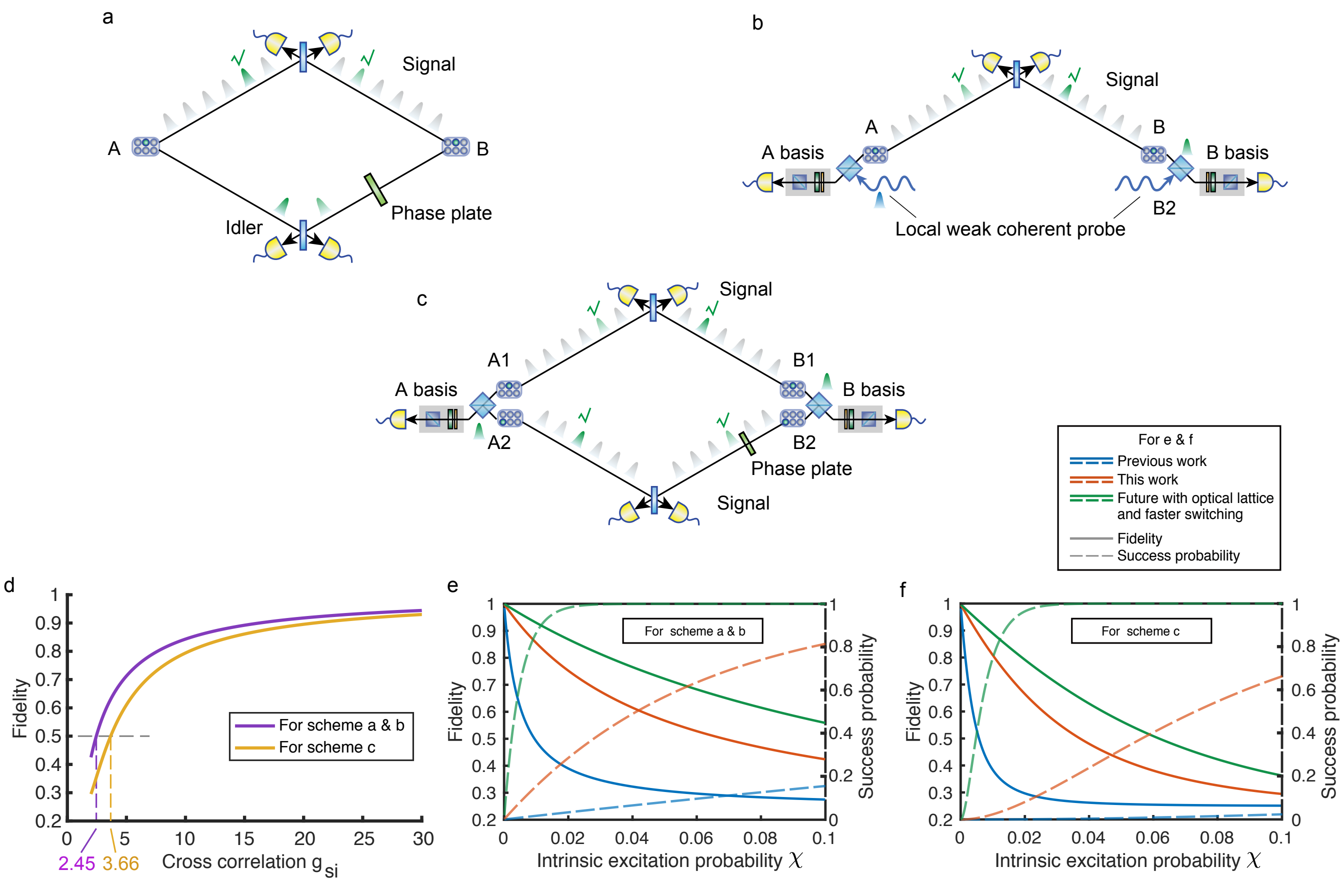

Supplementary Fig. 11. **Three schemes for generation and verification of distant atom-atom entanglement based on the atom-photon quantum correlation in this work.** **a**, In scheme a, we generate one pair of Fock-state atom-atom entanglement between repeter nodes A and B by interfering the signal photons from each node on a beamsplitter, and verify the entanglement by interfering two idler photon modes on another beamsplitter. Active stabilization of long idler channels is needed. **b**, In scheme b, by combining the idler photon with local weak coherent state, a Fock-state entanglement can be converted into a polarization entanglement for verification. **c**, In scheme c, we prepare two remote atom-atom entanglement between node A and B, then combine them to a polarization entanglement for verification. **d**, Simulated remote atom-atom entanglement fidelities in a function of cross-correlation g_{si} for each atom-photon quantum correlation, under the protocol and memory performance in this experiment, for the three different schemes demonstrated in a-c. For schemes a and b, the threshold to achieve a fidelity of 50% is $g_{si} > 2.45$. For scheme c, the threshold is 3.66. **e**, The fidelity and the cumulative success probability for remote atom-atom entanglement for schemes a and b, under the same 240 μs heralding protocol described in this experiment but with three different memory performances, as a function of a varying excitation probability χ. The solid (dashed) brown curve represents the fidelity (cumulative success probability) under the memory performance in this work, the solid (dashed) blue curve represents the fidelity (cumulative success probability) under the memory performance in [19], and the solid (dashed) green curve represents the fidelity (cumulative success probability) under the memory performance of a future setup with optical lattice array, optimized frequency conversion and optical paths, which is described in Supplementary Note 7. **f**, The fidelity and the cumulative success probability for remote atom-atom entanglement for schemes c, under the same heralding protocol described in this experiment but with three different memory performances same with e, as a function of a varying excitation probability χ.

$$\rho = \left(\frac{(g_{si}-1)^2}{g_{si}^2+1} - \chi\frac{8g_{si}(g_{si}-1)^2(g_{si}+1)}{(g_{si}^2+1)^2}\right)|\Psi\rangle\langle\Psi| + \left(\frac{2g_{si}}{g_{si}^2+1} + \chi\frac{8g_{si}(g_{si}-1)^2(g_{si}+1)}{(g_{si}^2+1)^2}\right)\rho_{\text{noise}} \tag{11}$$

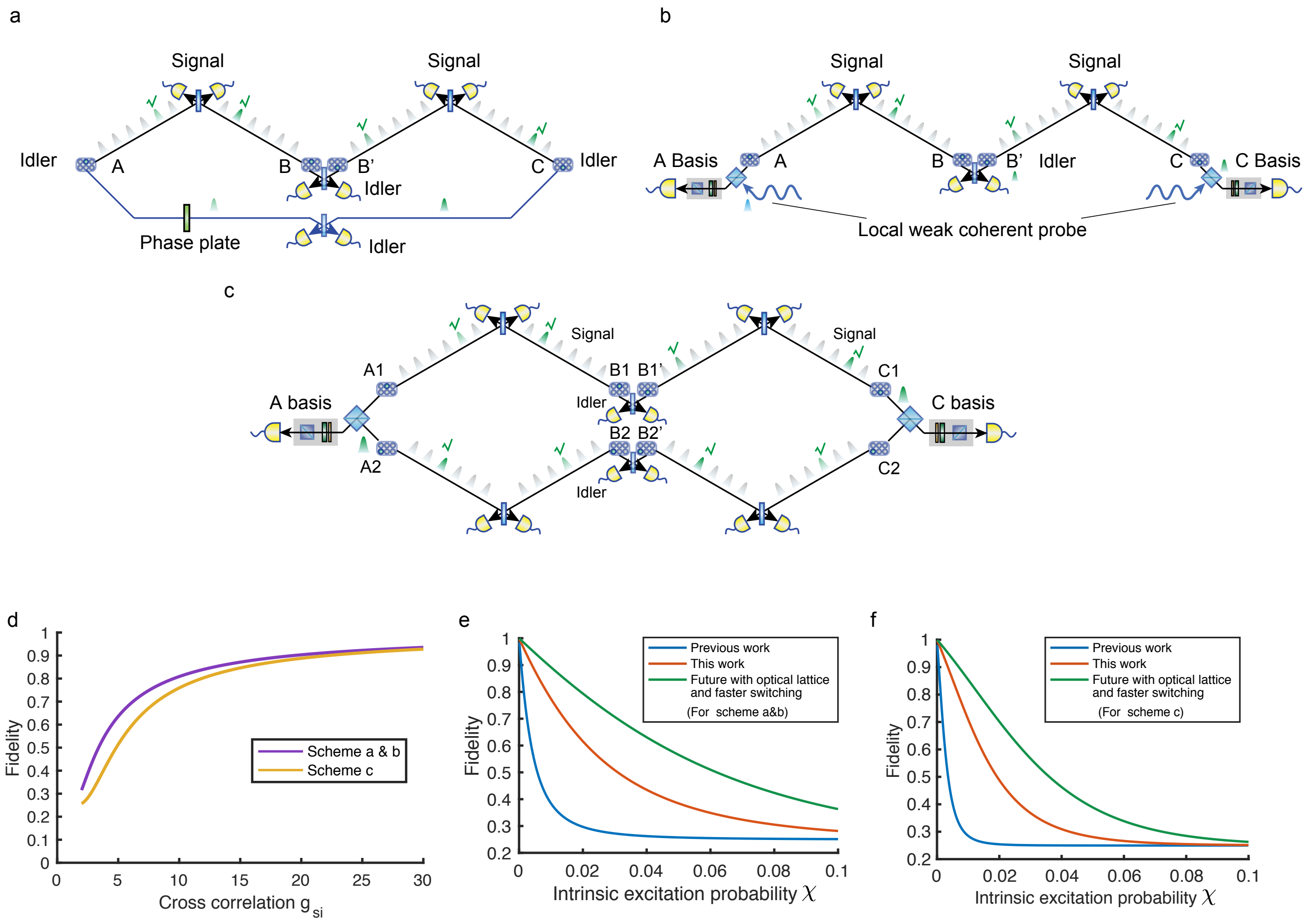

Supplementary Fig. 12. **Three schemes for generation and verification of distant atom-atom entanglement in a 3-node quantum repeater.** **a**, We prepare a Fock-state entanglement via a three-node quantum repeater protocol. Atom-atom entanglement between node A and C is prepared by three steps: (i) first generate atom-atom entanglement between node A and B via single photon interference, (ii) generate atom-atom entanglement between node B' and C, and (iii) entanglement swapping between B and B', which are different memory modes at node B. After the preparation of entanglement between node A and C, we can convert the memory modes into idler photons and interfere them on a beamsplitter in the middle to verify the Fock-state entanglement. **b**, Different to scheme a, after preparing the Fock-state entanglement between A and C, we can combine the idler photon with local weak coherent state and measure the fidelity of polarization encoded entanglement. **c**, One can also prepare two Fock-state entanglements between A and C, and combine them into a polarization entanglement to measure the fidelity. **d**, The fidelity versus the cross-correlation, under the condition in this experiment and three different schemes described in a-c. Here we consider a simplified case that all the atom-photon quantum correlations have the same g_{si}. **e-f**, The fidelity of the atom-atom entanglement between node A and C in a three-node quantum repeater protocol, in different schemes described in a-c, and under different memory performances. Here we assume the read out time is at the end of the protocol, thus the fidelity demonstrated here is a lower bound.

The fidelity of this density matrix is

$$F = Tr(|\Psi\rangle\langle\Psi|\rho) = 1 - \frac{3g_{si}}{2(g_{si}^2+1)} - \chi\frac{6g_{si}(g_{si}-1)^2(g_{si}+1)}{(g_{si}^2+1)^2} \quad (12)$$

The simulated atom-atom (idler-idler) entanglement fidelity under different cross correlation g_{si} (in each atom-photon quantum correlation) is shown in Supplementary Fig. 11d. We also demonstrate the simulated atom-atom entanglement fidelity and the atom-atom entanglement success probability as a function of different excitation probability χ, under different entangling schemes and memory performances, for a task to combine two 12 km atom-photon correlation into a 24 km atom-atom entanglement with the same excitation and heralding protocol in this work, as shown in Supplementary Fig. 11e and 11f. For simplicity, here we have assume the noise of frequency conversion is negligible in Supplementary Fig. 11e and Fig. 11f.

In addition, we also investigate the entanglement fidelity of a 3-node quantum repeater which connects two elementary atom-

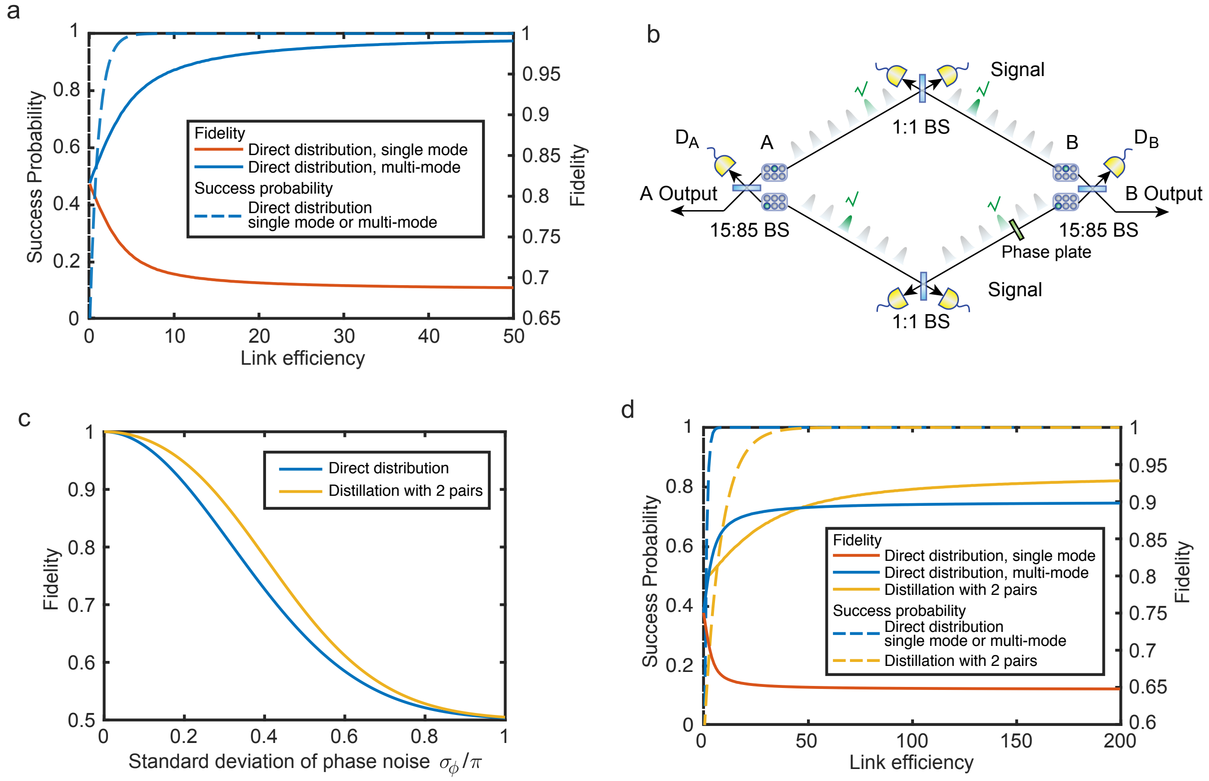

Supplementary Fig. 13. **Performance advantage through multi-mode quantum repeater and entanglement distillation.** **a**, The success probability (dashed curve) and fidelity (solid curves) for the entanglement distribution task, under two different cases (single-mode or multi-mode quantum repeater) described in Supplementary Note 10. **b**, The scheme for entanglement distillation. **c**, The atom-atom entanglement fidelity as a function of phase noise. Here we assume a phase noise of Gaussian distribution with standard deviation of σ_ϕ. **d**, The success probability (dashed curves) and fidelity (solid curves) for the entanglement distribution task with a single-mode repeater, multi-mode repeater, and entanglement distillation in a multi-mode repeater, as described in Supplementary Note 10.

atom entanglement links via entanglement swapping. The schemes for entanglement verification is similar to the verification of atom-atom entanglement generated in an elementary link as discussed above and illustrated in Supplementary Fig. 12. Here we still use the three schemes to verify entanglement of 3-node repeater, which are implemented by remote idler photon interference on a beamsplitter (Supplementary Fig. 12a), combination with local weak coherent pulses (Supplementary Fig. 12b), and combining two Fock-state entanglement into a polarization entanglement (Supplementary Fig. 12c). We demonstrate the entanglement fidelity as a function of the cross correlation g_{si} (Supplementary Fig. 12d), and different excitation probability χ under various memory performances (Supplementary Fig. 12e and 12f). For simplicity, here we have assume the noise of frequency conversion is negligible in Supplementary Fig. 12e and Fig. 12f.

Supplementary Note 10. Performance advantage via multi-mode quantum repeater node

In this section we demonstrate two schemes to improve the fidelity of remote entanglement via the use of multi-mode quantum repeater nodes in the future, compared with the scheme where a single-mode memory is used. We compare their performances in a task of generating a remote atom-atom entanglement in an elementary link, during a time interval equal to memory coherence time $T_{\rm coh}$ (which is different from the 240 μs protocol in this work), and analyze their performances. Here we assume an ideal condition that the multi-mode quantum repeater are working at a very high link efficiency, and the entanglement establishing attempts can run continuously in parallel with other operations, which can be realized in the future. Here we demonstrate two schemes with multi-mode quantum repeater node which can achieve a better performance over single-mode quantum repeater in this task.

The first scheme is based on large capacity of a multi-mode quantum memory. Instead of heralding and storing only one pair of atom-atom entanglement in a single-mode quantum repeater, a multi-mode quantum repeater can herald and store many pairs of atom-atom entanglement in different pairs of memory modes. With this redundancy in resource, one can use the most recently

generated atom-atom entangled pair for further network applications or entanglement swapping tasks to expand the remote entanglement to larger scale. In this way, the later generated atom-atom entanglement suffers from a shorter waiting time, thus less decoherence and higher fidelity can be achieved. The single-mode case can also be viewed as selecting the first generated entanglement in a multi-mode case (multi-mode selects the last one), and the cumulative probabilities of generating an entanglement are the same (Supplementar Fig. 13a). We illustrate the simulated fidelity of the delivered atom-atom entanglement in Supplementary Fig. 13a. We can see that with the multi-mode quantum repeater node and selecting the last generated entangled pair, the fidelity of the delivered entanglement increases with link efficiency. This is due to the expected time cost of generating an entanglement decreases with larger link efficiency, thus the last generated entangled pair suffers from less decoherence. One can also see that with larger link efficiency, the single-mode case has a lower fidelity. This is because with larger link efficiency, the generating time of the entanglement is more close to the beginning, thus the entanglement suffers from more decoherence during the storage. As the single-mode memory cannot store more than one remote entanglement, the entanglement generation attempts have to be halted after one pair of entanglement is heralded. Thus, although both of the case can have a probability close to unity for generating an entanglement with large link efficiency, only the multi-mode case can achieve a high fidelity, with the use of the last generated entanglement to alleviate the influence of decoherence.

The second scheme is to use entanglement distillation to improve the fidelity of the distributed entanglement. Here we use a distillation scheme based on [20], and illustrated in Supplementary Fig. 13b. In this scheme, one needs to first generate two pairs of remote atom-atom entanglements between A and B, and then interfere the retrieved idler photon on two 85/15 beamsplitters. Once either detector D_A or D_B clicks, the output idler entangled pair is distilled to a state of higher fidelity. Here, to better simulate the real-world scenario, we add phase noise originated from experimental imperfections into consideration. The fidelity of generated remote entanglement and distilled entanglement under different phase noise is shown in Supplementary Fig. 13c. Here we assume the generated entanglement has an initial fidelity of 90% due to experimental noise. We consider such a distillation scheme, that each time two new atom-atom entanglements are heralded, we pair them together and read them out to generate one distilled entanglement at the end of the protocol. Due to the finite read out efficiency, the distillation protocol can sometimes fail. If there are many successfully distilled entanglement, we use the newest one as the final output. We illustrate the fidelity and success probability of three cases in Supplementary Fig. 13d, which include single-mode repeater, the multi-mode repeater (select the last generated entanglement) without distillation, and the multi-mode repeater with distillation. It is shown that though the distillation scheme, one can generate a remote atom-atom entangled pair with higher fidelity than no distillation case.

Here we also explain why the data collection can be accelerated by reading out at most 3 excited memory modes as shown in Fig. 4 and Fig. 5. The data collection means the record of the signal-idler photon coincidence, thus we need to collect as many signal-idler photon coincidence in each 240 μs protocol. In a 240 μs protocol, sometimes more than one signal photons can be recorded from different memory modes, thus we can read out all these excited memory modes to idler photons to accelerate this process. For example, if the excitation in two memory modes are herald by recording corresponding signal photons during the heralding stage, we can read out both of them to idler photons one by one. By this scheme we can collect signal-idler coincidences two times faster in this 240 μs protocol of excitation and heralding, compared to the scheme of only reading one of them (and discard other excited modes). In both Fig. 4a and Fig. 5a, we demonstrate the read out of two modes in a single 240 μs run. In the situation that the p_{total} is large (for example, $p_{\text{total}} = 0.47$), the chance of heralding more than one modes is not negligible. Thus by this scheme we can accelerate the data collection rate.

Another consideration is that, creating many copies of entanglement between many pairs of memory modes is an advantage of multimode quantum repeater. To exploit this advantage, the ability of reading out many excited memory modes in a programmed way is necessary, and is demonstrated partly in this way. Here the different quantum correlations between different pairs of signal photon and memory modes are independent to each other, all of these can be used for further applications. When the excitation probability gets high, for example, for link efficiency close to unity, the chance of creating more than one excitation is high. Since all of these generated correlations are useful in later application, it is natural all of them should be measured, to demonstrate the ability to individually control each excited memory mode, and to accelerate the data collection at the same time.

Supplementary Note 11. Success probability of generating remote atom-atom entanglement through single-photon interference

In this section we explain the reason why the success probability is $2p_c$ if we combine a pair of atom-photon quantum correlations with success probability of p_c into one atom-atom entanglement via single photon interference. This can also explain why the link efficiency doubles in this process. We also analyze why the distance doubles in atom-atom entanglement ($2L$) compared to atom-photon correlation (L).

The configuration of the single photon interference is illustrated in Supplementary Fig. 14. Here in each attempt, the generated atom-photon quantum state for A and B can be expressed as

$$|\psi\rangle_{a,S_A} = \sqrt{1-p_c}|00\rangle_{a,S_A} + \sqrt{p_c}a^\dagger S_A^\dagger|00\rangle_{a,S_A} \tag{13}$$

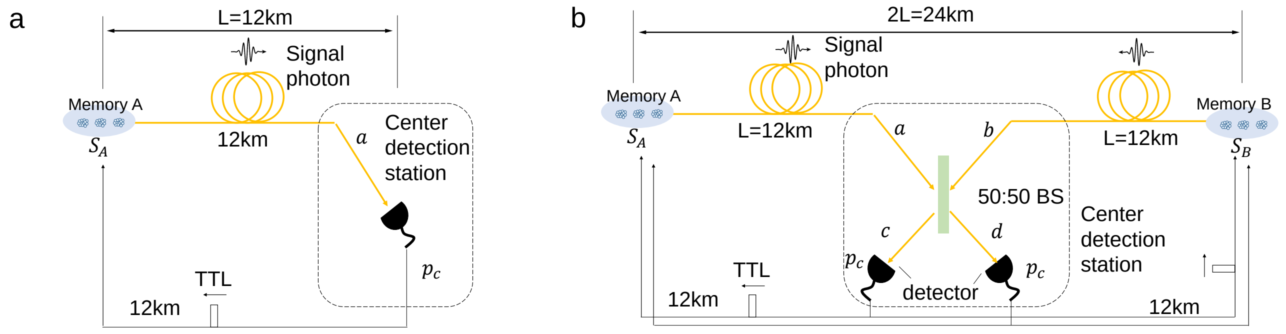

Supplementary Fig. 14. **Success probability and entanglement distance of generating remote atom-atom entanglement through single-photon interference. a**, The protocol of generating a heralded atom-photon quantum correlation (this experiment). We excite atom-photon correlation in node A locally, and send the signal photon to the middle detection station over a $L = 12\,\mathrm{km}$ fiber, and is detected by the detector. The heralding (success) probability is p_c for the state in Supplementary Equation (13). Then the detector click is sent back to node A over another 12 km fiber, and we read out the excited memory mode after we receive the TTL from the detection center. The whole protocol costs $\frac{2L}{c} = 120\,\mu\mathrm{s}$. **b**, The protocol of generating an atom-atom entanglement by combining two atom-photon correlations via single photon interference. Here one excites atom-photon correlations in repeater node A and B at the same time, and send the signal photons over two different 12 km fibers to the detection station in the middle for interference. After the interference, a click on either of the two detectors at the two outputs of the beamsplitter can herald a successful generation of atom-atom entanglement. Each detector has a chance of p_c to detect a photon, thus the total heralding (success) probability is $2p_c$, if the state of the two atom-photon from node A and B can be expressed as Supplementary Equation (13) and (14) (combine two atom-photon correlations). Then the center detection station sends the heralding TTL to both nodes A and B over the 12 km fibers. Node A and B can read out the excited modes after receiving TTLs. This protocol also costs $2L/c = 120\,\mu\mathrm{s}$ as in the atom-photon correlation, and have a success probability of $2p_c$. The atom-atom entanglement distance $2L = 24\,\mathrm{km}$ is also twice of the atom-photon correlation $L = 12\,\mathrm{km}$, if node A, the detection center, and node B are on the same line.

and

$$|\psi\rangle_{b,S_B} = \sqrt{1-p_c}|00\rangle_{b,S_B} + \sqrt{p_c}b^\dagger S_B^\dagger|00\rangle_{b,S_B} \tag{14}$$

Here $a^\dagger$ is the creation operator of signal mode from node A, $S_A^\dagger$ is the creation operator of memory mode of node A, p_c is the success (heralding) probability of each atom-photon correlation generation attempt, $b^\dagger$ is the creation operator of signal mode from node B, and $S_B^\dagger$ is the creation operator of memory mode of node B. Here we have ignored the higher excitations as $p_c \ll 1$. With the atom-photon quantum state in Supplementary Eq. (13) and Supplementary Eq. (14), the success probability of generating a heralded atom-photon quantum correlation following the protocol of our experiment is p_c in each attempt for either A and B. For simplicity but without loss of generality, here we assume that p_c contains all the inefficiencies in the channel and the detector has a unity efficiency. In the experiment, we use a single detector to replace the beamsplitter and two detectors (Supplementary Fig. 14a) to measure the atom-photon correlation of node A. Note that the success probability with either one detector (p_c) or the combination of one beamsplitter and two detectors ($\frac{p_c}{2}$ for each detector and the sum is p_c) are the same. The joint state of the both sides is

$$|\psi\rangle_{a,b,S_A,S_B} = (1-p_c)|0000\rangle_{a,b,S_A,S_B} + \sqrt{p_c(1-p_c)}(a^\dagger S_A^\dagger|0000\rangle_{a,b,S_A,S_B} + b^\dagger S_B^\dagger|0000\rangle_{a,b,S_A,S_B}) + O(p_c) \tag{15}$$

In the single photon heralded entangling scheme, the two signal modes a and b from each side are sent into two input channels of a beamsplitter, as shown in Supplemenary Fig. 14. After interference on the beamsplitter, two output modes c and d are each sent into a detector. The transformation between the input and output modes of a 50:50 beamsplitter can be expressed as

$$a^\dagger \to \frac{c^\dagger + d^\dagger}{\sqrt{2}} \qquad b^\dagger \to \frac{c^\dagger - d^\dagger}{\sqrt{2}} \tag{16}$$

Combining Supplementary Eq. (15) and Supplementary Eq. (16), we have

$$|\psi\rangle_{c,d,S_A,S_B} = (1-p_c)|0000\rangle_{c,d,S_A,S_B} + \sqrt{p_c(1-p_c)}(\frac{c^\dagger + d^\dagger}{\sqrt{2}}S_A^\dagger + \frac{c^\dagger - d^\dagger}{\sqrt{2}}S_B^\dagger)|0000\rangle_{c,d,S_A,S_B} + O(p_c) \quad (17)$$

$$= (1-p_c)|0000\rangle_{c,d,S_A,S_B} + \sqrt{p_c(1-p_c)}(c^\dagger \frac{S_A^\dagger + S_B^\dagger}{\sqrt{2}} + d^\dagger \frac{S_A^\dagger - S_B^\dagger}{\sqrt{2}})|0000\rangle_{c,d,S_A,S_B} + O(p_c) \quad (18)$$

$$= (1-p_c)|vac\rangle + \sqrt{p_c(1-p_c)}|1\rangle_c \frac{|10\rangle_{S_AS_B} + |01\rangle_{S_AS_B}}{\sqrt{2}} + \sqrt{p_c(1-p_c)}|1\rangle_d \frac{|10\rangle_{S_AS_B} - |01\rangle_{S_AS_B}}{\sqrt{2}} + O(p_c) \quad (19)$$

Here $|vac\rangle$ is vacuum state, $|1\rangle_c$ or $|1\rangle_d$ is one photon in the c or d mode, $|1\rangle_{S_A}$ or $|1\rangle_{S_B}$ is one excitation in the memory mode at node A or B.

We can clearly see that from Supplementary Eq. (19), in the case of $p_c \ll 1$ (p_c is at the level of 0.1% in our experiment), we have a probability of $p_c(1-p_c) \approx p_c$ to record a photon on the detector in the c mode, and a Fock-state atom-atom entanglement $\frac{|10\rangle_{S_AS_B}+|01\rangle_{S_AS_B}}{\sqrt{2}}$ is heralded. The detector for d mode can also record a photon with a success probability of p_c, which heralds an atom-atom entanglement $\frac{|10\rangle_{S_AS_B}-|01\rangle_{S_AS_B}}{\sqrt{2}}$. Therefore, this single photon interference succeeds with a probability of $p_c + p_c = 2p_c$, with a click on either detector can herald a remote atom-atom entanglement. Since the memory coherence time T_{coh} is same in both the atom-photon quantum correlation generation and the atom-atom entanglement generation, the link efficiency of the atom-atom entanglement should be twice the value in atom-photon correlation. Here we prove this quantitatively. In this case, the link efficiency for heralded atom-photon quantum correlation generation over a distance L is (see Eq. (1))

$$\eta_{\text{link, a-p}} = \frac{T_{\text{coh}}}{T_{\text{ent, a-p}}} = \frac{T_{\text{coh}}}{\frac{2L}{cNp}} = \frac{c}{2L} T_{\text{coh}} N p_c \quad (20)$$

Here the success probability $p = p_c$ in atom-photon correlation generation. Other symbols are defined in Eq. (1). This is also the link efficiency measured in this experiment.

The link efficiency for heralded atom-atom entanglement when combining two atom-photon correlations by single photon interference over a distance $2L$ (see Supplementary Fig. 14) is

$$\eta_{\text{link, a-a}} = \frac{T_{\text{coh}}}{T_{\text{ent, a-a}}} = \frac{T_{\text{coh}}}{\frac{2L}{cNp}} = \frac{c}{2L} T_{\text{coh}} N (2p_c) \quad (21)$$

Here $p = 2p_c$ is the heralding (success) probability of the atom-atom entanglement in each trial, as discussed above. We can see that the success probability doubles in atom-atom entanglement, but the time cost of each attempt is still the same $\frac{2L}{c}$ (Supplementary Fig. 14). Thus the link efficiency of atom-atom entanglement is twice the value of the atom-photon correlation

$$\eta_{\text{link, a-a}} = 2\eta_{\text{link, a-p}} \quad (22)$$

The entanglement distance also doubles when we combine two heralded atom-photon quantum correlations to generate a remote atom-atom entanglement. As shown in Supplementary Fig. 14, the heralded atom-photon correlation is distributed over a fiber length of $L = 12$ km (the distance between the signal photon a and the memory S_A, which is also the distance between node A and the center detection station). When two such atom-photon correlation (a and S_A, b and S_B) is combined by interfering their signal photon in a center detection station in the middle, the heralded atom-atom entanglement between S_A and S_B is separated by $12 \times 2 = 24$ km, as shown in Supplementary Fig. 14.

Finally, we also prove that the atom-atom entanglement $\eta_{\text{link, a-a}}$ is twice the expected number of heralded atom-photon correlation generated within memory coherence time over L. The link efficiency for atom-photon quantum correlation is

$$\eta_{\text{link, a-p}} = \frac{T_{\text{coh}}}{T_{\text{ent, a-p}}} = T_{\text{coh}} r_{\text{ent, a-p}} \quad (23)$$

Here $r_{\text{ent, a-p}} = \frac{1}{T_{\text{ent, a-p}}}$ is the generation rate of the heralded atom-photon correlation. $T_{\text{coh}} r_{\text{ent, a-p}}$ is thus the expected number of heralded atom-photon correlation generated within memory coherence time. Since $\eta_{\text{link, a-p}} = T_{\text{coh}} r_{\text{ent, a-p}}$ (Supplementary Equation (23)), $\eta_{\text{link, a-p}}$ is the expected number of heralded atom-photon correlation generated within memory coherence time. As $\eta_{\text{link, a-a}} = 2\eta_{\text{link, a-p}}$, then $\eta_{\text{link, a-a}}$ is twice the expected number of heralded atom-photon correlation generated within memory

coherence time over L.

---

[1] Zhang, S., Shi, J., Cui, Z., Wang, Y., Wu, Y., Duan, L. & Pu, Y. Realization of a programmable multi-purpose photonic quantum memory with over-thousand qubit manipulations. Preprint at https://arxiv.org/abs/2311.10292 (2023).

[2] Pu, Y.-F., Jiang, N., Chang, W., Yang, H.-X., Li, C. & Duan, L.-M. Experimental realization of a multiplexed quantum memory with 225 individually accessible memory cells. *Nat. Commun.* **8**, 15359 (2017).

[3] Wang, Y.-F., Li, J.-F., Zhang, S.-C., Su, K.-Y., Zhou, Y.-R., Liao, K.-Y., Du, S.-W., Yan, H. & Zhu, S.-L. Efficient quantum memory for single-photon polarization qubits. *Nat. Photon.* **13**, 346 (2019).

[4] Gujarati, T. P., Wu, Y.-K. & Duan, L.-M. Intrinsic retrieval efficiency for quantum memories: A three-dimensional theory of light interaction with an atomic ensemble. *Phys. Rev. A* **97**, 033826 (2018).

[5] Humphreys, P. C., Kalb, N., Morits, J. P., Schouten, R. N., Vermeulen, R. F., Twitchen, D. J., Markham, M. & Hanson, R. Deterministic delivery of remote entanglement on a quantum network. *Nature* **558**, 268-273 (2018).

[6] Liu, J.-L., Luo, X.-Y., Yu, Y., Wang, C.-Y., Wang, B., Hu, Y., Li, J., Zheng, M.-Y., Yao, B., Yan, Z., Teng, D., Jiang, J.-W., Liu, X.-B., Xie, X.-P., Zhang, J., Mao, Q.-H., Jiang, X., Zhang, Q., Bao, X.-H. & Pan, J.-W. A multinode quantum network over a metropolitan area. Preprint at https://arxiv.org/abs/2309.00221 (2023).

[7] Lago-Rivera, D., Grandi, S., Rakonjac, J. V., Seri, A. & de Riedmatten, H. Telecom-heralded entanglement between multimode solid-state quantum memories. *Nature* **594**, 37-40 (2021).

[8] Zhou, L., Lin, J.-P., Jing, Y.-M. & Yuan, Z.-L. Twin-field quantum key distribution without optical frequency dissemination *Nat. Commun.* **14**, 928 (2023).

[9] Pu, Y.-F., Wu, Y.-K., Jiang, N., Li, C., Zhang, S. & Duan, L.-M. Experimental entanglement of 25 individually accessible atomic quantum interfaces. *Sci. Adv.* **4**, eaar3931 (2018).

[10] Pogorelov, I. *et al.* Compact Ion-Trap Quantum Computing Demonstrator. *PRX Quantum* **2**, 020343 (2021).

[11] Yu, Y. *et al.* Entanglement of two quantum memories via fibres over dozens of kilometres. *Nature* **578**, 240 (2020).

[12] Krutyanskiy, V., Galli, M., Krcmarsky, V., Baier, S., Fioretto, D. A., Pu, Y., Mazloom, A., Sekatski, P., Canteri, M., Teller, M., Schupp, J., Bate, J., Meraner, M., Sangouard, N., Lanyon, B. P. & Northup, T. E. Entanglement of trapped-ion qubits separated by 230 meters. *Phys. Rev. Lett.* **130**, 050803 (2023).

[13] Collins, O. A., Jenkins, S. D., Kuzmich, A. & Kennedy, T. A. B. Multiplexed Memory-Insensitive Quantum Repeaters. *Phys. Rev. Lett.* **98**, 060502 (2007).

[14] Duan, L.-M., Lukin, M. D., Cirac, J. I. & Zoller, P. Long-distance quantum communication with atomic ensembles and linear optics. *Nature* **414**, 413-418 (2001).

[15] Sangouard, N., Simon, C., de Riedmatten, H. & Gisin, N. Quantum repeaters based on atomic ensembles and linear optics. *Rev. Mod. Phys.* **83**, 33-80 (2011).

[16] Farrera, P., Maring, N., Albrecht, B. Heinze, G. & de Riedmatten, H. Nonclassical correlations between a C-band telecom photon and a stored spin-wave. *Optica* **3**, 1019 (2016).

[17] Chou, C. W., de Riedmatten, H., Felinto, D., Polyakov, S. V., van Enk, S. J. & Kimble, J. Measurement-induced entanglement for excitation stored in remote atomic ensembles. *Nature* **438**, 828 (2005).

[18] Chou, C. W., Laurat, J., Deng, H., Choi, K. S., de Riedmatten, H., Felinto & Kimble, J. Functional Quantum Nodes for Entanglement Distribution over Scalable Quantum Networks. *Science* **316**, 1316 (2007).

[19] Chang, W., Li, C., Wu, Y.-K., Jiang, N., Zhang, S., Pu, Y.-F., Chang, X.-Y. & Duan, L.-M. Long-Distance Entanglement between a Multiplexed Quantum Memory and a Telecom Photon. *Phys. Rev. X* **9**, 041033 (2019).

[20] Salart, D., Landry, O., Sangouard, N., Gisin, N., Herrmann, H., Sanguinetti, B., Simon, C., Sohler, W., Thew, R. T., Thomas, A. & Zbinden, H. Purification of Single-Photon Entanglement. *Phys. Rev. Lett.* **104**, 180504 (2010).
